# Primary monophasic synovial sarcoma of the chest wall in an adolescent: a case report

**DOI:** 10.1186/s13256-026-06155-z

**Published:** 2026-05-30

**Authors:** Grace Tannous, Mohammad Alaa Aldakak, Omar Al Ayoubi, Mohammad Hesso, Yamama Abo Dakka, Joudy Eljerdy, Mohamad Ali Nahas, Hussain Chaban

**Affiliations:** 1 Faculty of Medicine, Damascus University, Damascus, Syrian Arab Republic; 2 Al Assad University Hospital, Damascus University, Damascus, Syrian Arab Republic

**Keywords:** Synovial sarcoma, Chest wall tumor, Adolescent, Soft tissue sarcoma, Case report

## Abstract

**Background:**

Synovial sarcoma is a rare malignant mesenchymal tumor, accounting for 5–10% of soft tissue sarcomas, mainly affecting adolescents and young adults near large joints. Thoracic involvement, particularly in the chest wall, is extremely rare. Diagnosis is difficult due to histologic overlap with other tumors, requiring immunohistochemistry and molecular detection for confirmation. Treatment involves complete surgical excision with adjuvant therapy, guided by a multidisciplinary approach.

**Case presentation:**

A 15-year-old Arab male presented with right upper limb numbness, pain, and progressive dyspnea. Physical examination revealed a firm, non-tender supraclavicular mass above the right clavicle, with mild edema of the right upper extremity. Chest radiography showed a right apical opacity with mediastinal shift to the left. Contrast-enhanced computed tomography (CT) demonstrated a large, heterogeneous mass originating from the right upper chest wall, extending into the lower cervical region, displacing the trachea, encasing the subclavian vein, and infiltrating adjacent neural and muscular structures. CT-guided biopsy confirmed a diagnosis of monophasic synovial sarcoma. The patient underwent complete surgical excision with vascular reconstruction, followed by referral for adjuvant oncologic therapy.

**Conclusions:**

Primary chest wall synovial sarcoma is a rare and diagnostically challenging tumor. Early recognition, accurate histopathological confirmation, and multidisciplinary management with complete surgical resection are essential to optimize outcomes and reduce the risk of recurrence or metastasis.

## Introduction

Soft tissue sarcomas (STS) comprise a heterogeneous group of tumors and represent fewer than 1% of all malignancies in adults [1]. Classical synovial sarcoma (SS) is a rare malignant soft tissue tumor of mesenchymal origin that exhibits histological characteristics of both carcinoma and sarcoma [1]. It constitutes a distinct subtype of soft tissue sarcomas (STS), accounting for 5–10% of all cases, and is known for its tendency to affect younger individuals and its distinctive clinical presentation [2]. These tumors most commonly arise near joint regions in the extremities, such as the upper limbs, thigh, knee, ankle, and foot; however, thoracic synovial sarcoma is a rare variant that can originate in the chest wall, pleura, lungs, heart, or mediastinum [3]. The symptoms result from either direct invasion of the chest wall by the tumor or compression of adjacent underlying structures [3]. Diagnosing SS can be challenging due to its morphological similarity to other tumors, making immunohistochemical markers like transducer-like enhancer of split-1 (TLE-1) essential for confirmation [4, 5]. Accurate data on the incidence in the chest wall remain limited, as only a few cases have been documented in the literature, often with insufficient follow-up [6]. Best practice guidelines emphasize that a definitive diagnosis should be established or confirmed by a histopathologist with specific expertise and regular experience in evaluating soft tissue tumors before initiating further management [7]. Accordingly, the clinical and surgical management of synovial sarcoma should be multidisciplinary and tailored to the individual patient, with complete tumor excision with negative margins being the primary treatment goal, typically followed by chemotherapy and adjuvant therapy [8].

In this case, we present a 15-year-old male with a rare monophasic synovial sarcoma arising in the right upper thoracic wall, manifesting with upper limb neurological symptoms and progressive dyspnea, ultimately requiring complex multidisciplinary surgical resection.

## Case presentation

**Fig. 1:**
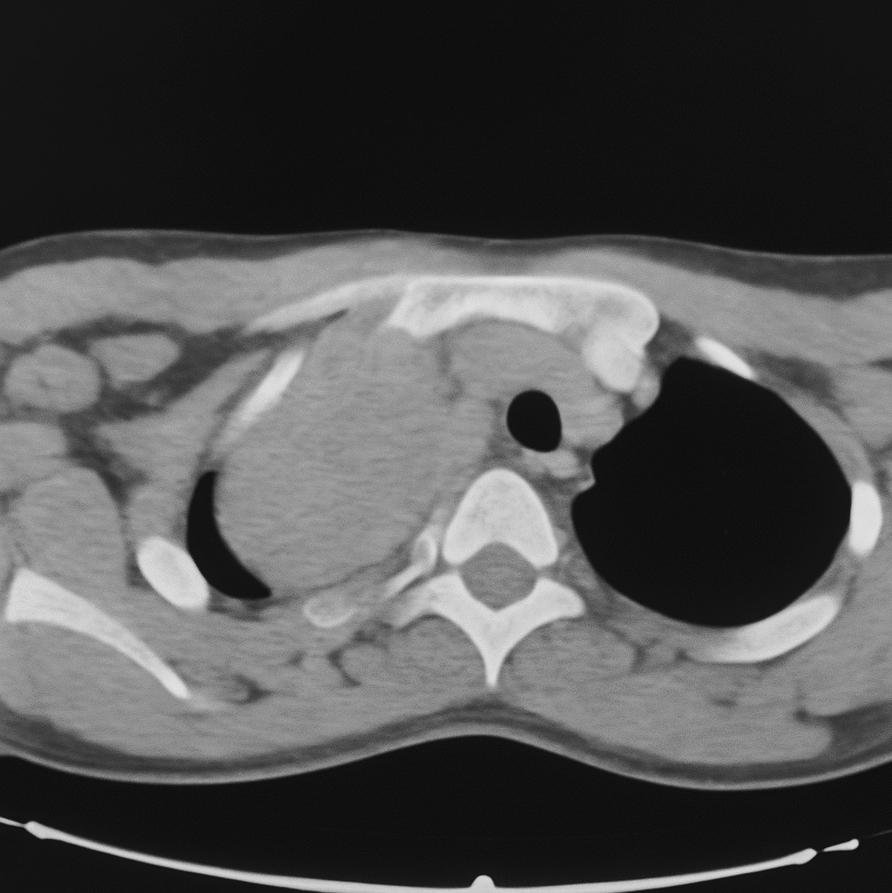
Axial contrast-enhanced CT of the upper thorax shows a well-defined heterogeneous mass originating from the right upper chest wall, displacing the trachea and mediastinum leftward and partially compressing the adjacent lung

**Fig. 2:**
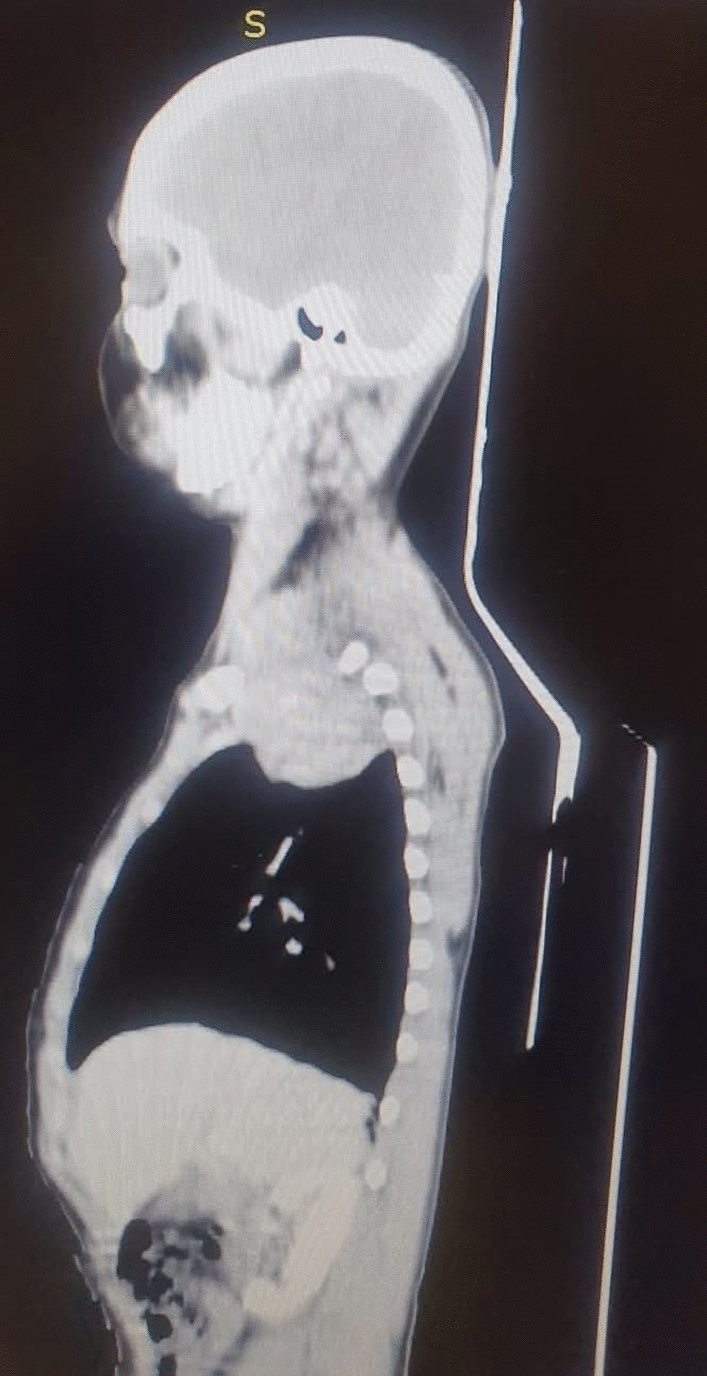
Sagittal contrast-enhanced CT of the head, neck, and thorax shows a vertically oriented, mixed-density mass extending from the right upper chest wall to the lower cervical region, displacing the trachea anteriorly and compressing adjacent structures, with intact spinal column

**Fig. 3:**
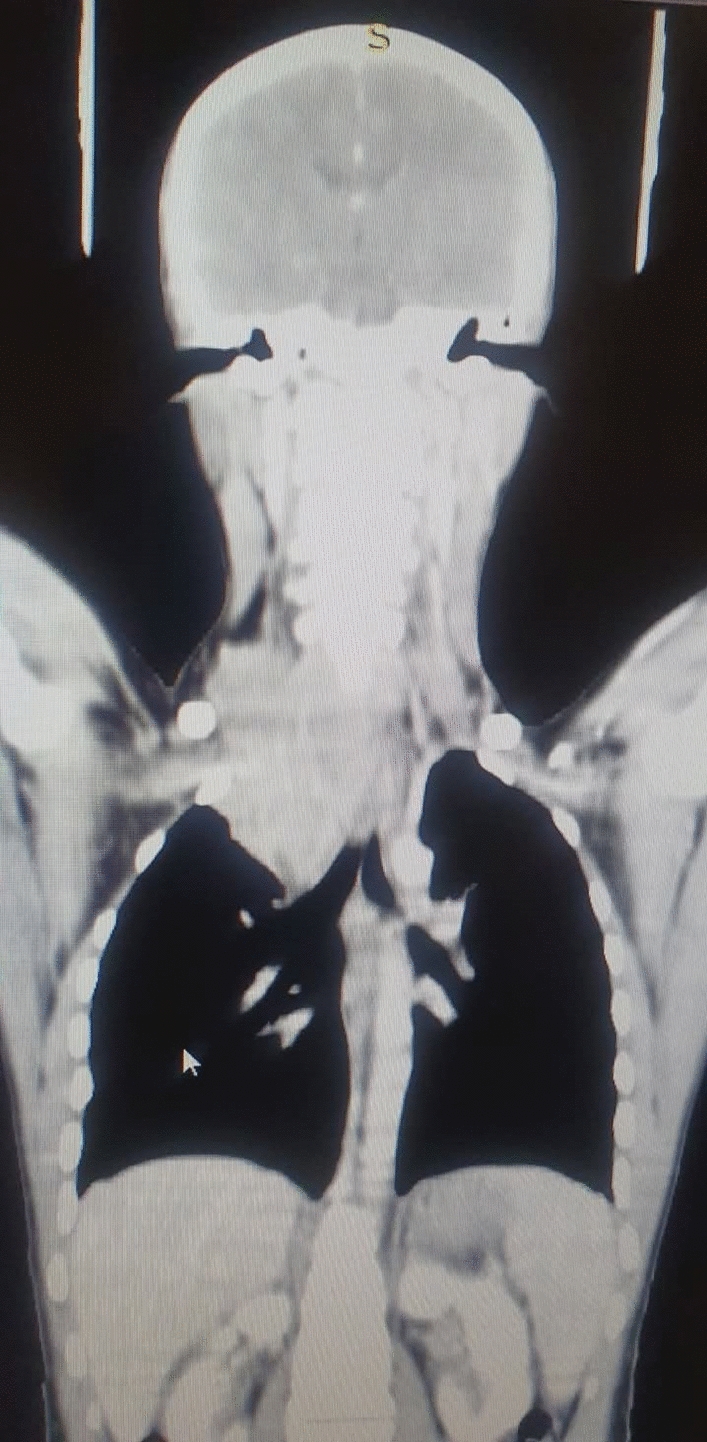
Coronal CT of the head, neck, and thorax shows a right-sided chest wall mass extending upward into the cervical region, displacing the trachea leftward and compressing mediastinal and pulmonary structures. The lesion has irregular borders and heterogeneous attenuation

A 15-year-old Arab male patient presented with complaints of right upper limb numbness and pain, accompanied by progressive dyspnea. The patient was previously healthy with no significant past medical or surgical history. Family history was negative for malignancy or genetic disorders. Psychosocial history was unremarkable, with normal developmental and functional status prior to presentation. Physical examination revealed a firm, non-tender mass in the right supraclavicular region above the right clavicle, with associated mild edema of the right upper extremity. The overlying skin was intact with no signs of ulceration. Neurological examination of the right upper limb demonstrated reduced sensation. Chest radiography (X-ray) demonstrated a right apical opacity with significant mediastinal shift to the left. A subsequent contrast-enhanced Computed tomography (CT) scan of the neck and chest revealed a large, heterogeneous soft tissue mass arising from the right upper chest wall, extending superiorly into the lower cervical region (Figs. 1, 2 and 3). The lesion measured approximately 6 × 8 cm and demonstrated heterogeneous enhancement with areas of internal necrosis, without evidence of calcification or bony destruction.

The mass displaced the trachea to the left and encased the right subclavian vein, with close proximity to the subclavian artery. It also infiltrated adjacent structures, including the phrenic and vagus nerves as well as the scalene muscles. Based on clinical and radiological findings, the tumor was consistent with locally advanced disease (stage III).

Histopathological analysis following CT-guided biopsy confirmed the diagnosis of monophasic spindle synovial sarcoma grade 2, characterized by spindle cell mesenchymal proliferation, composed of relatively uniform elongated cells arranged in intersecting fascicles. The tumor cells exhibited scant cytoplasm, elongated nuclei with finely dispersed chromatin, and inconspicuous nucleoli. Mitotic figures were identified, although not markedly elevated. Focal areas of tumor necrosis were present, consistent with the radiological findings.

Immunohistochemistry supported the diagnosis with positivity for CD99, TLE-1, and negativity for Myogenin, CD34, FLI-1, and ERG, unfortunately molecular testing for the SS18-SSX gene fusion was not performed due to limited availability of molecular diagnostic techniques at our institution.

**Fig. 4:**
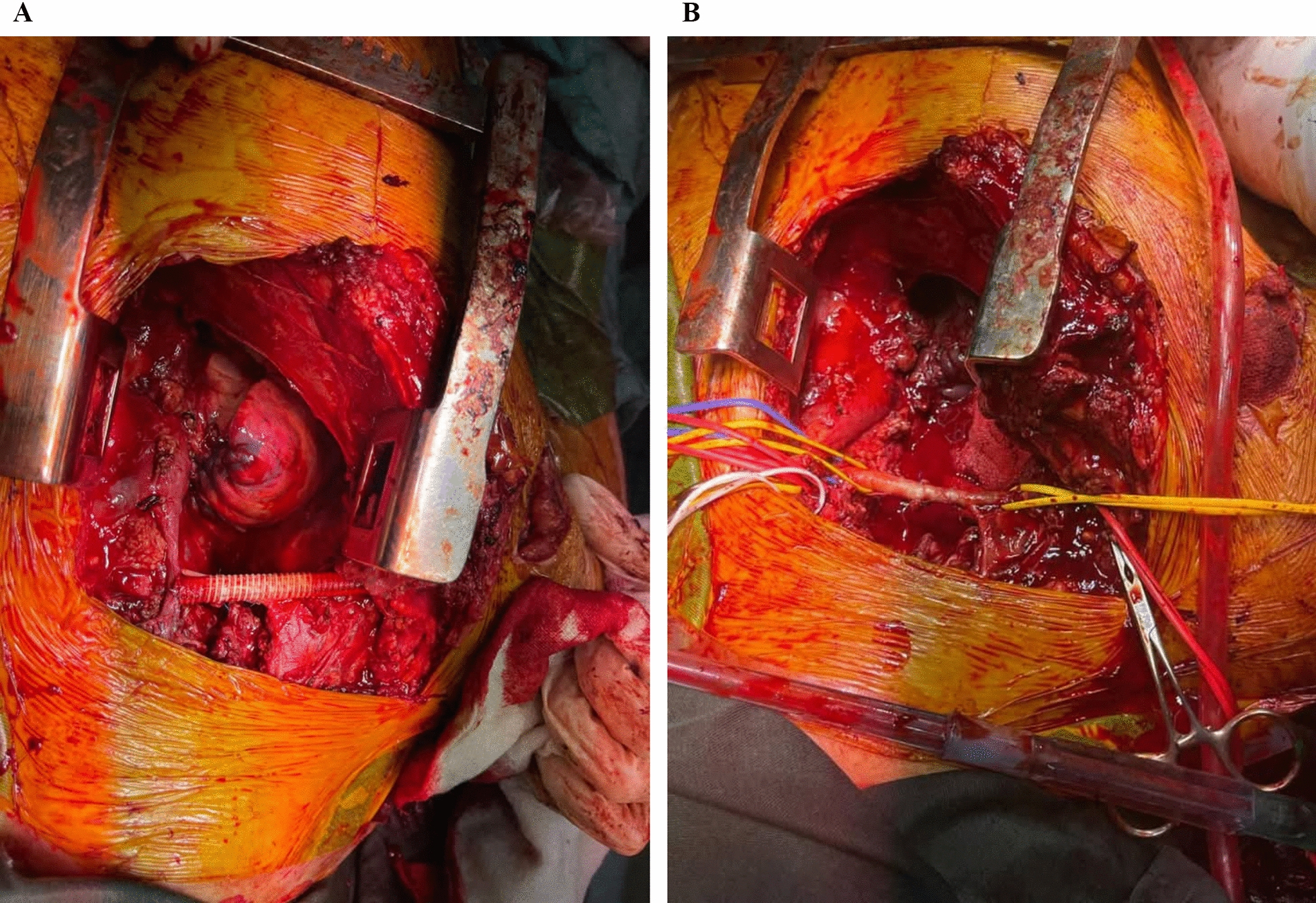
**a** Intraoperative photo showing the surgical field after en bloc resection of a chest wall synovial sarcoma. A synthetic graft replaces the resected right subclavian vein, with meticulous dissection of surrounding structures, highlighting tumor infiltration and complex vascular reconstruction. **b** Intraoperative view showing dissected vascular structures post-tumor resection. Vascular loops and colored markers isolate the subclavian artery and vein, with careful preservation of nearby nerves, illustrating meticulous dissection and preparation for reconstruction

Due to the tumor’s anatomical complexity and proximity to major vascular and neural structures, the patient was referred for surgical intervention. A multidisciplinary surgical team from the departments of cardiothoracic and vascular surgery performed a wide *en bloc* resection of the tumor along with adjacent involved tissues (Fig. 4a, b).

Intraoperative findings confirmed tumor infiltration into the subclavian vascular bundle and surrounding nerves. Careful dissection was performed to preserve vital structures where possible. Due to vascular involvement, reconstruction of the right subclavian vein was performed using a reinforced synthetic graft to ensure venous patency. This approach was undertaken to achieve complete tumor excision with negative margins while preserving critical neurovascular structures. Surgical resection margins were reported as free of tumor, confirming complete excision, focal areas of tumor necrosis were present, consistent with the radiological findings. Postoperative imaging confirmed successful tumor resection, with resolution of mediastinal deviation and restoration of tracheal position. The postoperative course was uneventful, and the patient was discharged in stable condition.

Following complete surgical excision, the patient was referred to a tertiary oncology center, where adjuvant chemotherapy consisting of ifosfamide and doxorubicin was administered every 3 weeks for a total of 6 cycles (over 5 months). The treatment was well tolerated, with no significant adverse events, in accordance with established treatment guidelines. At 6-month follow-up, the patient remained in good general condition, with no clinical or radiological evidence of tumor recurrence or distant metastasis. Long-term follow-up is ongoing to monitor for late recurrence. To better illustrate the sequence of clinical events and management steps, a structured timeline summarizing the patient’s clinical course from symptom onset to follow-up is presented in Table 1.

**Table 1:** Timeline of events

Time	Event
T0 (symptom onset)	The patient initially presented with progressive right upper limb numbness, pain, and dyspnea
T0 + 2–3 weeks	Clinical examination revealed a firm right supraclavicular mass. Chest radiography demonstrated a right apical opacity with mediastinal shift to the left
T0 + 3–4 weeks	Contrast-enhanced CT scan of the neck and chest showed a large heterogeneous right upper chest wall mass extending into the lower cervical region with compression and displacement of adjacent mediastinal structures. CT-guided biopsy confirmed monophasic synovial sarcoma
T0 + 5–6 weeks	The patient underwent multidisciplinary surgical management, including wide *en bloc* resection of the tumor with involvement of adjacent neurovascular structures and reconstruction of the right subclavian vein using a synthetic graft
Postoperative period (immediate)	The postoperative course was uneventful, and the patient was discharged in stable condition
Follow-up period	The patient was referred to a tertiary oncology center and received adjuvant chemotherapy. At 6-month follow-up, he remained clinically stable with no evidence of recurrence or distant metastasis

## Discussion

Synovial sarcoma, first described in 1893 [4], represents a unique subtype within soft tissue sarcomas (STS) accounting for about 6% of all STS cases [9]. While it is considered the most common non-rhabdomyosarcomatous STS in children and adolescents—responsible for approximately 30% of cases in patients younger than 20 years old [10]—it may develop across all age groups [11], with no significant gender predilection [12].

While synovial sarcomas typically develop near joints, tendons, or bursae—with approximately 80% of cases occurring in the lower limbs, particularly around the knees [13] Thoracic synovial sarcoma is an extremely rare entity that can arise in various intrathoracic structures, including the chest wall, pleura, lungs, heart, and mediastinum, with only a limited number of cases reported in the literature [14–16]. Within this spectrum, primary synovial sarcoma of the chest wall is particularly uncommon and has been reported only in a small number of cases [3, 13, 16].

Although synovial sarcoma may be misdiagnosed as benign due to its slow growth and asymptomatic presentation in early stages, it is in fact malignant and associated with a poor prognosis [13]. In addition, synovial sarcomas can present with a variety of symptoms, such as palpable lumps, painful masses, motor or sensory disturbances, indolent swellings, or pain without any detectable mass [17]. Specifically, when involving the chest wall, symptoms vary according to the structures compressed or invaded by the tumor, with patients commonly presenting with chest pain, cough, dyspnea, decreased breath sounds, and weight loss [18]. Previously reported cases of chest wall synovial sarcoma commonly present as large, locally aggressive masses with invasion into adjacent structures, often posing significant diagnostic and therapeutic challenges [4, 19]. In our patient, right upper limb numbness and progressive dyspnea were consistent with compression of major neurovascular and mediastinal structures, as described in previously reported cases. However, this case is particularly notable for its superior cervical extension and marked encasement of major vascular structures, features that are rarely reported in the literature.

Baseline imaging in the diagnostic process generally includes ultrasound (US), magnetic resonance imaging (MRI), computed tomography (CT), and occasionally plain radiographs [20].

On CT, chest wall synovial sarcoma typically appears as a heterogeneously enhancing mass with well-defined margins, accompanied by cortical bone destruction, calcifications, and invasion into the chest wall musculature [14, 21]. Consistent with these typical imaging features, the CT scans of our patient revealed a heterogeneous mass that displaced the trachea and invaded adjacent vascular and neural structures.

Possible differential diagnoses on imaging include peripheral nerve sheath tumors and solitary fibrous tumors; however, these entities cannot be reliably distinguished from synovial sarcoma based solely on imaging features, making histopathological evaluation essential for confirming the exact diagnosis. Synovial sarcomas exhibit three cellular patterns: monophasic (composed of spindle cells), biphasic (containing both spindle and epithelial cells), and poorly differentiated types. Among these, the monophasic variant is the most frequent, representing 50–60% of cases, with a predominance of spindle cell components [19].

Similar to other soft tissue sarcomas, the diagnosis of synovial sarcoma is challenging to confirm solely based on histological features, and it becomes even more difficult in cases lacking clear biphasic differentiation. Therefore, immunohistochemical analysis is essential, typically revealing neoplastic cells with diffuse immunoreactivity for CK, EMA, Vimentin, Bcl-2, Actin, TLE-1, and CD99, alongside focal immunoreactivity for S-100 protein, while showing negativity for CD34 and Desmin [4, 5, 22]. While our patient, histopathological analysis revealed a monophasic synovial sarcoma with spindle cell morphology, and immunohistochemistry confirmed the diagnosis with positivity for CD99, TLE-1, and negativity for Myogenin, CD34, FLI-1, and ERG. Although molecular confirmation through detection of the SS18-SSX gene fusion is considered highly specific for synovial sarcoma, immunohistochemical markers such as TLE-1 are widely used and demonstrate high sensitivity in supporting the diagnosis when interpreted in conjunction with histopathological features [2, 23].

The treatment of choice for chest wall synovial sarcoma, as with other soft tissue sarcomas, involves a multimodal approach combining wide to radical surgical resection, radiation therapy, and adjuvant chemotherapy following surgery. This strategy is critical given the tumor’s high recurrence and metastasis risk, underscoring the need for vigilant follow-up [18, 24, 25]

In our patient, due to the tumor’s extensive local invasion and proximity to critical neurovascular structures, an *en bloc* surgical resection was performed followed by adjuvant therapy, aligning with current treatment guidelines.

This rare case of thoracic synovial sarcoma underscores the diagnostic difficulty associated with its unusual location and vague clinical presentation. In addition, the tumor’s deep invasion into vital neurovascular structures highlights the importance of meticulous surgical planning and coordinated multidisciplinary management.

## Conclusion

Thoracic wall synovial sarcoma, though rare, presents significant diagnostic and therapeutic challenges due to its aggressive nature, atypical symptoms, and proximity to critical structures. Successful management requires accurate histopathological confirmation, careful surgical planning, and coordinated multidisciplinary treatment. Reporting such uncommon presentations is essential to increase clinician awareness, enable earlier diagnosis, and guide tailored treatment strategies.

### Limitations

This case report has several limitations that should be acknowledged. First, molecular confirmation of synovial sarcoma through detection of the SS18–SSX gene fusion could not be performed due to limited availability of molecular diagnostic facilities in our institution. Second, magnetic resonance imaging (MRI), which is considered a valuable modality for detailed local staging and soft tissue characterization, was not available in our setting at the time of evaluation. These limitations reflect resource constraints in our healthcare system and may have affected the extent of preoperative diagnostic assessment. Finally, the follow-up period remains relatively short, and longer surveillance is required to better assess long-term outcomes and disease recurrence.

## Data Availability

All data generated or analyzed during this case report are included in this published article.
